# A Switch between Antioxidant and Prooxidant Properties of the Phenolic Compounds Myricetin, Morin, 3′,4′-Dihydroxyflavone, Taxifolin and 4-Hydroxy-Coumarin in the Presence of Copper(II) Ions: A Spectroscopic, Absorption Titration and DNA Damage Study

**DOI:** 10.3390/molecules24234335

**Published:** 2019-11-27

**Authors:** Klaudia Jomová, Lenka Hudecova, Peter Lauro, Miriama Simunkova, Saleh H. Alwasel, Ibrahim M. Alhazza, Marian Valko

**Affiliations:** 1 Department of Chemistry, Constantine the Philosopher University in Nitra, 949 74 Nitra, Slovakia; kjomova@ukf.sk (K.J.); lhudecova@ukf.sk (L.H.); plauro@ukf.sk (P.L.); 2 Faculty Chemical and Food Technology, Slovak University of Technology, 812 37 Bratislava, Slovakia; miriama.simunkova@stuba.sk; 3 King Saud University, Zoology Department, College of Science, Riyadh 11451, Saudi Arabiaimalhazza@hotmail.com (I.M.A.)

**Keywords:** flavonoids, hydroxyl groups, copper, antioxidant, prooxidant, DNA damage

## Abstract

The beneficial effects of polyphenols, predominantly in the context of oxidative stress-related diseases such as cancer, cardiovascular diseases and neurological conditions including Alzheimer’s and Parkinson’s diseases, have been documented by a number of papers and reviews. The antioxidant/prooxidant properties of phenolic compounds are related mainly to the number and positions of hydroxyl groups and to their redox metal (Cu, Fe) chelating capacity. In this work we studied structurally distinct phenolic molecules such as myricetin, morin, 3′,4′-dihydroxy-flavone, taxifolin and 4-hydroxycoumarin, either alone or as interacting with Cu^2+^ ions. EPR and UV-Vis spectroscopy confirmed that the effective binding of cupric ions to phenolic compounds requires the presence of the 3-OH and 4-CO groups on the flavonoid C ring and unsaturated C2-C3 bond of the C-ring, which permits through-conjugation with the B-ring. An ABTS assay revealed that radical scavenging activities of phenolic compounds are related to their number of hydroxyl groups, planarity of the molecular skeleton, extent of delocalization and they decrease in the order: myricetin > morin > 3′,4′-dihydroxyflavone ~ 4-hydroxy coumarin > taxifolin. Absorption titrations indicate that copper ions can modulate the DNA binding affinity of flavonoids via the formation of their Cu-chelates. Gel electrophoresis measurements indicated that the protective effect of the phenolic compounds decreases in the order: 3′,4′-dihydroxyflavone > 4-OH coumarin > morin > taxifolin ~ myricetin. This can be explained by the fact that myricetin, taxifolin and morin form stable Cu(II) complexes capable of causing DNA damage via interaction with DNA and ROS formation via the Fenton reaction. Application of ROS scavengers revealed the formation of singlet oxygen, superoxide and hydroxyl radicals and their concerted synergistic effect on the DNA. The overall results suggest that the most pronounced DNA damage has been observed for flavonoids containing higher number of hydroxyl groups (including 3-OH group of the C ring), such as myricetin (six hydroxyl groups), morin and taxifolin (five hydroxyl groups) in the presence of Cu(II) ions. The proposed mechanism of action by which Cu(II) complexes of myricetin, morin and taxifolin interact with DNA predispose these substances to act as potential anticancer agents. The anticancer activity of phenolic compounds can be explained by their moderate prooxidant properties, which can boost ROS formation and kill cancer cells. Alternatively, slight prooxidant properties may activate antioxidant systems, including antioxidant enzymes and low molecular antioxidants such as glutathione and thus act as preventive anticancer agents.

## 1. Introduction

Owing to their potential beneficial health effects, polyphenolic compounds have attracted significant attention during the past two decades [1]. The most plentiful group of polyphenol compounds that occurs in Nature is the flavonoids. Flavonoid biosynthesis is stimulated by light and therefore these compounds accumulate mainly in the peel and bark of plants, but also in their seeds and flowers. In plants, the primary function of flavonoids is to provide protection against reactive oxygen species (ROS) that are produced during the process of photosynthesis [2].

The beneficial health effects of flavonoids have been associated with their ability to prevent oxidative damage to living organisms, caused by ROS and/or reactive nitrogen species (RNS) [3], and it is believed that one of the most important mechanisms associated with the beneficial effects of these natural pigments and the prevention of variety of diseases is their ability to function as antioxidants. The beneficial effects of polyphenols have been documented predominantly in the context of oxidative stress-related diseases such as cancer, and also neurological conditions including Alzheimer’s and Parkinson’s diseases [4]. In addition to their antioxidant properties, flavonoids may confer further beneficial consequences, including digestive stimulation, and anti-inflammatory, anti-microbial, anti-viral and anti-mutagenic effects [5,6,7].

**Figure 1:**
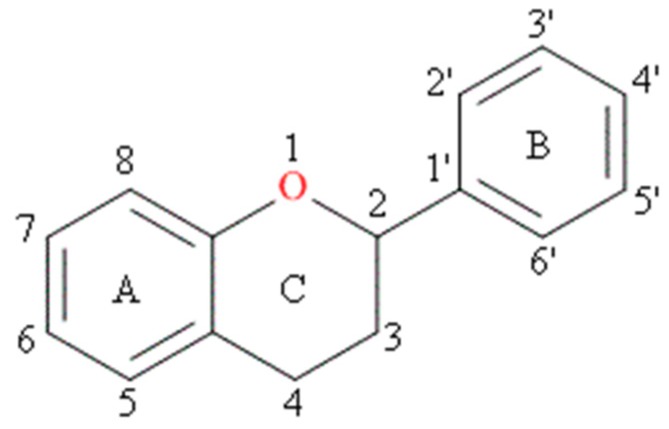
Basic skeleton of flavonoids.

Although more than 4000 flavonoids are currently known [8], it is quercetin, kaempferol, myricetin, apigenin and luteolin that have been reported as important components in human diets (as present in fruits, vegetables, cereals) [9]. The characteristic feature of each flavonoid molecule is a skeleton composed of 15 carbon atoms which contains two aromatic rings, A and B, linked together by a three-carbon bridge that is part of a heterocyclic, pyran ring C (Figure 1).

Due to their great variety, flavonoids are categorized according to structural characteristics (oxidation level, substituents) into multiple classes [10]. They may also exist as glycosides, which, in contrast to aglycones, possess bound carbohydrate groups at positions 3 or 7, or they may contain methyl groups. The hydroxyl groups of flavonoids can be in positions 3, 5, 7, 2′, 3′, 4′ and 5′, which give these compounds the properties of weak acids, which can deprotonate to form anionic species, depending both on their pKa, and the pH of the local environment.

**Figure 2:**
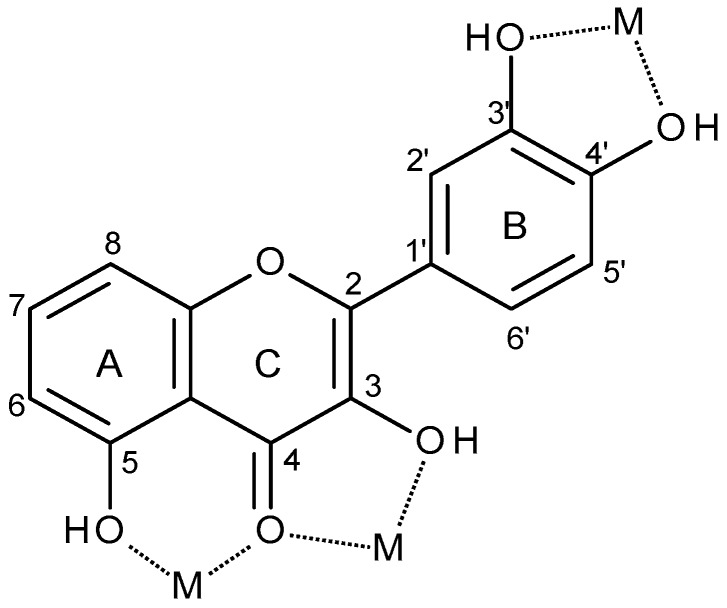
Typical metal (M) chelation sites in flavonoids.

Flavonoids play an important role in the bioavailability of metal ions which are present in trace amounts in the body, and can also detoxify heavy metals such as chromium or lead: the latter being due to the ability of their hydroxyl groups to chelate free redox metal ions and form stable complexes, which are subsequently eliminated from the organism [11]. Molecules of the specific group of flavonols have three main sites where transition metal ions can potentially be coordinated: (i) the 3-hydroxyl and the 4-carbonyl groups in the C ring, (ii) the 5-hydroxyl group of the A ring and the 4-carbonyl group in the C ring and (iii) the 3′- and 4′-hydroxyl groups of the B ring (Figure 2) [7,12,13,14].

The position and number of particular functional hydroxyl groups in flavonoids and their interaction with redox ions such as iron or copper have several important consequences:

(i) A flavonoid molecule that contains multiple hydroxyl groups typically exhibits a greater antioxidant (radical scavenging) capacity than a flavonoid with a smaller number of hydroxyl groups [15,16]. (ii) Transition metal ions such as copper or iron catalyze the decomposition of hydrogen peroxide, resulting in the formation of damaging hydroxyl radicals, via the Fenton reaction [17]:

Fe^2+^/Cu^+^ + H_2_O_2_ → Fe^3+^/Cu^2+^ + OH^−^ + ^•^OH (1)

Metal ions coordinated/chelated by flavonoids [18,19,20,21,22] usually exhibit a suppressed catalytic activity in the Fenton reaction and hence a reduced formation of hydroxyl radicals. However, many flavonoids also have reducing properties and when coordinated to copper(II) they may (partially) reduce the metal ion (i.e., Cu^2+^ → Cu^+^) which causes them to be more active as catalysts in the Fenton reaction [15]. There are thus two competing effects in that interaction of metal ions with flavonoids: metal-chelation effect of flavonoids suppresses catalytic activity of metal ions in the Fenton reaction, while alternatively, reduction of metal ions to lower oxidation states, due to the reducing capacity of flavonoids, results in the augmentation of hydroxyl radical formation. (iii) Complexes of transition metal ions with flavonoids are effective SOD mimetic compounds and are thus capable of affecting the redox state of cells [20,21]. (iv) Redox metals coordinated with flavonoids may exert redox cycling mechanism resulting in the formation of ROS (prooxidant behaviour) [18]. (v) The majority of flavonoid molecules have essentially flat structures with rigid rings that contain a system of conjugated double bonds and those flavonoids coordinated to redox ions may intercalate into DNA and cause damage (prooxidant behaviour) [18].

**Figure 3:**
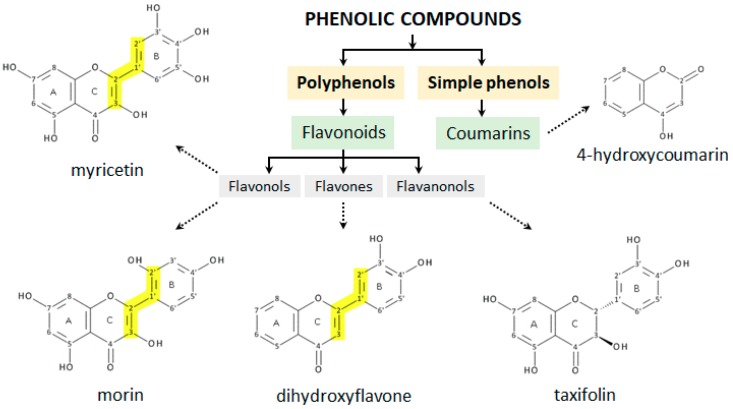
Structure of phenolic compounds used in the study based on their categorization into classes. The conjugation of A/C-rings to a polyhydroxyphenyl moiety at the C-2 position is marked in yellow.

From the above discussion, it follows that in the presence of redox ions, flavonoids may behave either as antioxidants or as prooxidants. Since the results obtained to date are not fully consistent, due to the complexity of the systems investigated, and the various experimental conditions employed, the aim of the present work is to study structurally distinct phenolic molecules such as myricetin, morin, 3′,4′-dihydroxyflavone, taxifolin and 4-hydroxycoumarin (Figure 3), either alone or as interacting with Cu^2+^ ions. The flavonoids were selected with the aim to determine and contrast the effect of the number and positions of the hydroxyl groups on the copper chelating efficiency and antioxidant vs prooxidant activity, paying particular attention to elucidating those structural characteristics of the compounds studied which influence a switch from antioxidant to prooxidant behaviour [23].

Copper(II) ions were chosen for interaction studies with flavonoids on the basis of the biological importance of copper as an integral element of metalloenzymes, in the synthesis of neurotransmitters, in the origin of oxidative stress in Alzheimer’s and Parkinson’s diseases, cancer diseases and in other conditions [4]. Those pathological states of an organism that are characterized by free (unbound) copper, which can catalyze formation of ROS via the Fenton reaction and consequently cause damage to all important biomolecules including DNA are of great clinical importance [15]. Thus, we see that in Alzheimer’s disease, which is characterized by a significantly raised content of copper in amyloid plaques, therapeutic approaches based on a dietary supplementation with flavonoids that are capable of chelating free copper, resulted in an ameliorated progression of those symptoms that are typical for the condition. From this and other examples of pathological states of an organism that are characterized by a malfunctioning copper metabolism, it follows that it is worthwhile to study copper(II)-flavonoid interactions not only from a molecular structural point of view but also in a search for prospective flavonoid chelation therapies.

The nature of copper(II)-flavonoid interactions were studied using both EPR and UV-Vis spectroscopy, while the biological activity of flavonoids, both as free compounds, and as copper(II)-flavonoid chelates, was studied through their interactions with DNA, as determined using absorption titrations and gel electrophoresis.

## 2. Results and Discussion

The interaction of each of the phenolic compounds studied with cupric ions was determined using a combination of EPR and UV-Vis spectroscopic methods. Additionally, the effect of coordination with copper(II) ions on the antioxidant properties of the phenolic compounds was studied using ABTS assays; the interaction of the phenolic compounds and of their copper complexes with DNA, along with DNA protective role was studied by means of a combination of absorption titrations and gel electrophoresis.

### 2.1. Chelation of Cu(II) by Phenolic Compounds

The interaction of cupric ions with phenolic compounds was studied in DMSO solution at 77 K using EPR spectroscopy and at room temperature by UV-Vis spectroscopy.

#### 2.1.1. EPR Spectroscopy of Copper(II)-Phenolic Complexes

**Figure 4:**
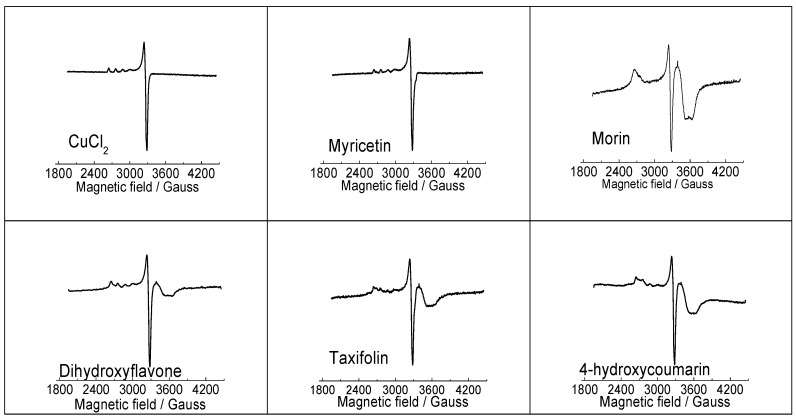
EPR spectra of CuCl_2_ and Cu(II) complexes (1:2) with myricetin, morin, 3′,4′-dihydroxyflavone, taxifolin and 4-hydroxycoumarin (1:2), recorded in DMSO solution at 77 K. EPR data: CuCl_2_ (g_┴_ = 2.083, g_‖_ = 2.414, A_‖_ = 118 Gauss); Myricetin (g_┴_ = 2.084, g_‖_ = 2.413, A_‖_ = 115 Gauss); Morin (g_┴_ = 2.085, g_‖_ = 2.521); Dihydroxyflavone (g_┴_ = 2.085, g_‖_ = 2.397, A_‖_ = 115 Gauss); Taxifolin (g_┴_ = 2.081, g_‖_ = 2.393, A_‖_ = 120 Gauss); 4-hydroxycoumarin (g_┴_ = 2.084, g_‖_ = 2.451, A_‖_ = 115 Gauss).

EPR spectroscopy is a sensitive physical technique that is used for the detection and determination of paramagnetic species in various coordination environments and because cupric ions (*d*^9^ configuration) contain one unpaired electron they can be studied using EPR [24]. EPR spectra of the CuCl_2_ reference system, along with selected Cu(II)-flavonoid complexes, all measured in their DMSO solutions at 77 K are presented in Figure 4. It is immediately clear that the EPR spectrum of Cu(II)-complex with myricetin is different from those recorded for Cu(II) interaction with morin, dihydroxyflavone, taxifolin and 4-hydroxycoumarin. The difference in the EPR spectra of Cu(II) interacting with morin, dihydroxyflavone, taxifolin and hydroxycoumarin is substantiated by the presence of an overshoot peak at about 3500–3600 Gauss, originating probably from an existence of aggregated Cu(II) species in DMSO solution upon cooling to 77 K. It appears that with the exception of myricetin, flavonoids with lower number of hydroxyl groups and reduced Cu(II) chelating capacity have greater tendency to aggregate upon cooling in DMSO solution. Considerably more informative were UV-Vis spectra discussed in the next section.

#### 2.1.2. UV-Vis Spectroscopy of Cu(II)-Phenolic Complexes

**Figure 5:**
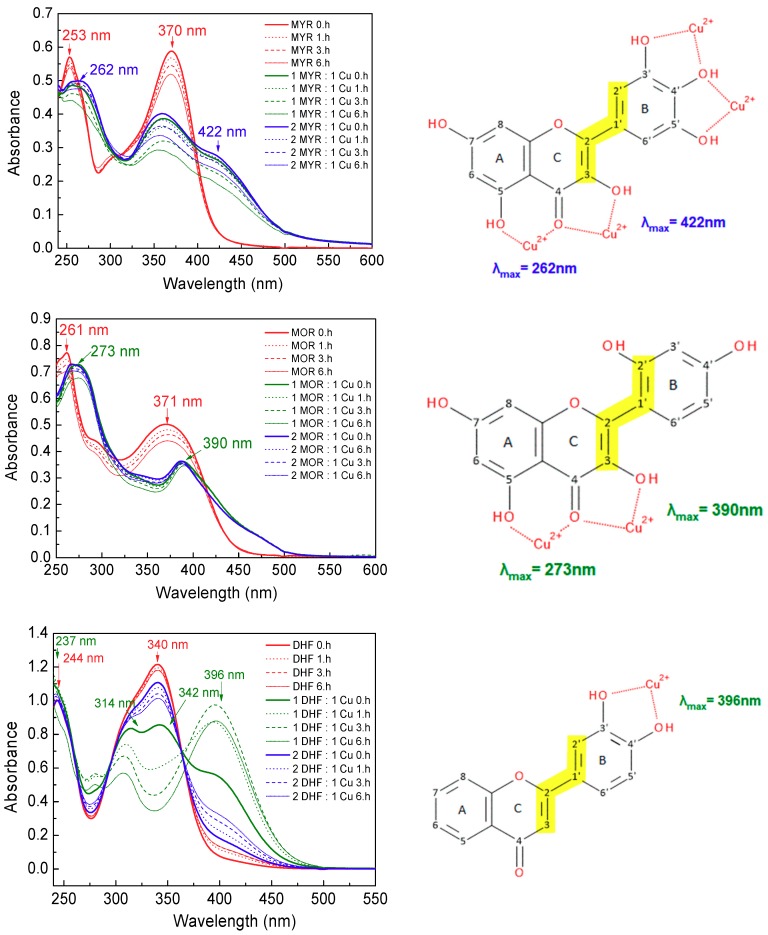
UV-Vis spectra of flavonols myricetin (MYR), morin (MOR) and 3′,4′-dihydroxyflavone (DHF) and their Cu(II) complexes in DMSO-phosphate buffer (5% *v*/*v*) at molar ratios Cu:flavonols = 1:1 a 1:2 recorded at the beginning of the reaction (up to 10 min), then after 1, 3 and 6 h. Potential Cu(II) chelating sites of myricetin, morin and 3′,4′-dihydroxyflavone are shown on the right panel.

**Figure 6:**
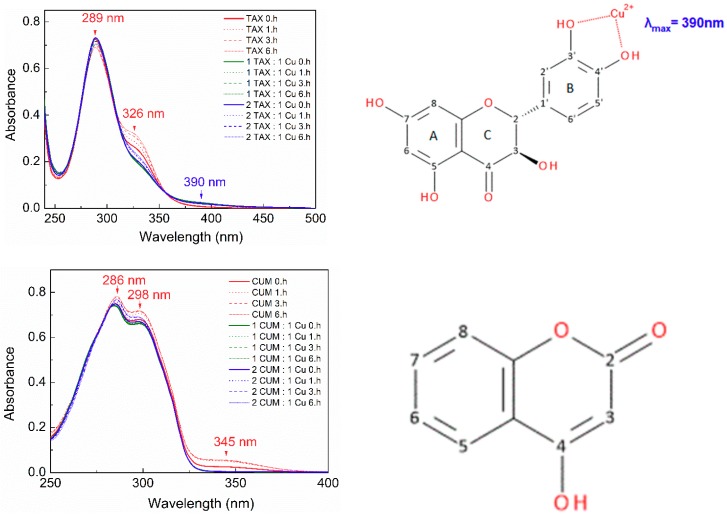
UV-Vis spectra of flavonols taxifolin (TAX) and simple phenol 4-hydroxycoumarin (CUM) and their Cu(II) complexes in DMSO-phosphate buffer (5% *v*/*v*) at molar ratios Cu: phenolic compound = 1:1 a 1:2 recorded at the beginning of the reaction (up to 10 min), then after 1, 3 and 6 h. at molar ratios Cu: phenolic compound = 1:1 a 1:2 recorded at the beginning of the reaction (up to 10 min), after 1, 3 and 6 h. Potential Cu(II) chelating sites of taxifolin and 4-hydroxycoumarin are shown on the right panel.

UV-Vis spectra can sensitively reflect interactions between the metal ions and organic molecules. The interaction of the phenolic compounds with cupric ions was evaluated on the basis of changes in the UV-Vis spectra of the free phenolic compounds, following addition of cupric ions at metal-to-phenolic compound molar ratios of 1:1 and 1:2. Spectra were recorded at four different times: at the beginning of the reaction (up to 10 min) and after 1 h, 3 h and then 6 h. Figure 5 shows the UV-Vis spectra of myricetin, morin and 3′,4′-dihydroxyflavone and their Cu(II) complexes. Figure 6 shows the UV-Vis spectra of taxifolin and 4-hydroxycoumarin and their Cu(II) complexes.

Generally, the UV-Vis spectra of both flavones and flavonols exhibit two characteristic absorption maxima related to the π → π* transitions localized within the A ring and ring C (~240–290 nm, referred to as band II, benzoyl system) and within the B-ring conjugated with the carbonyl of ring C (~300–415 nm, referred to as band I, cinnamoyl system) [25,26]. Complex formation between a phenolic compound (organic ligand) and metal ions (cupric ions) causes a marked bathochromic (red) shift of band I (up to 115 nm) toward the longer wavelengths due to the enhanced conjugative effect as a new ring is formed [27]. Following interaction of phenolic compounds with cupric ions, band II undergoes a mild red shift (up to 30 nm). Larger shifts of band I are linked with the participation of the cinnamoyl system in the complex formation, and larger shifts of band II reflect involvement of the benzoyl system in the formation of complexes between the phenolic compounds and the metal ions. To simulate the conditions close to the biological environment, the UV-Vis spectra of flavonoids interacting with Cu(II) ions were measured in DMSO-phosphate buffer (5% *v*/*v*).

The UV-Vis spectrum of myricetin (Figure 5, upper part) has two intense peaks at 253 nm (band II) and at 370 nm (band I). Following addition of cupric ions, the band II exhibits a small shift toward the longer wavelength (~9 nm), but the band I is split, causing a decrease in its intensity and a new absorption maximum is observed at 422 nm: this represents a red shift from the original peak of ~52 nm, which suggests participation of cinnamoyl ring system in the interaction of myricetin with cupric ions via its 3-OH and 4-CO groups (see upper scheme in Figure 5). Adjacent -OH groups of the B-ring in the myricetin molecule can provide an alternative site for the interaction with cupric ions. The lower intensity of the newly formed band at 442 nm suggests that some copper ions may also bind to myricetin through the 3′-,4′-,5′-OH groups of the catechol moiety (ring B).

The UV-Vis spectrum of morin (Figure 5 middle part) shows peaks centered at 261 nm (band II) and 371 nm (band I). When this flavonol is mixed with cupric ions, a slight shift occurs for the band II (~12 nm), but in contrast with the behaviour of myricetin, band I disappears and a new absorption band is observed at 390 nm (red shift about 19 nm). Both hydroxyl groups on ring B are separated from one other and therefore cannot act together to coordinate with cupric ions. The shift of band I results from a change of the electronic charge density in the cinnamoyl system containing the 3-OH group, which suggests that complex formation with Cu(II) has occurred through the 3-OH and 4-CO groups [27].

This type of interaction is supported by the greater proton acidity of the 3-OH group [28], as compared with the 5-OH group. The dissociation constants of phenolic -OH groups are influenced by the presence of the 4-CO group, which induces electronic density shifts via resonance effects [29]. The first complexation introduces a steric barrier that will hinder further complexation via the 4-CO and 5-OH groups [30]. Nevertheless, the comparable mild shifts of the band II towards higher wavelengths in the spectra of both flavonols (9 nm for myricetin and 12 nm for morin) indicate that an interaction of cupric ions through the 4-CO and 5-OH cannot be excluded.

Although the shift of band I is an indicator of interaction through the 3-OH and 4-CO groups for both myricetin and morin, the differences in the intensities of the new peaks observed at 422 nm for myricetin and at 390 nm for morin correlate with differences in their molecular structures. In the morin molecule, copper cannot bind to the OH groups of the B ring, so the 3-OH-4-CO groups most likely provide the only available site for interaction with cupric ions on the cinnamoyl part of the molecule: this is in agreement with the observation of a new absorption maximum at 390 nm along with the disappearance of the original band at 371 nm observed for the interaction of Cu(II) with morin.

The 3′,4′-dihydroxyflavone molecule possesses an isolated 4-CO group and also contains two -OH groups in the B-ring through which it can interact/complex with cupric ions. In the electronic absorption spectrum of 3′,4′-dihydroxyflavone (Figure 5, lower part), two peaks appear, at 244 nm (associated with the benzoyl skeleton) and at 340 nm (corresponding to the B-ring, cinnamoyl system). As it can be seen from Figure 5, the molar ratio of copper ions to this flavone significantly affects the shape of the spectrum. In the case of equimolar ratio of cupric ions and 3′,4′-dihydroxyflavone (green line in the spectrum), the first peak is slightly skewed to the lower wavelength (~7 nm) and the second, intense absorption maximum (340 nm) is split, causing a decrease in its intensity and a new absorption maximum is observed at 396 nm: this represents a red shift from the original peak of ~56 nm. Due to the absence of functional (-OH) groups in the C ring to be involved in binding copper ions, the only Cu(II) binding site in 3′,4′-dihydroxyflavone is characterised by the 3′-OH and 4′-OH groups in the B ring (see lower scheme in Figure 5).

Figure 5 also shows the change in band intensity over time. While in the spectrum recorded after 10 min (indicated as 0. hour) the intensity of the new band at 396 nm (indicating copper-flavone interaction) is weak, after 1 h its intensity increases significantly with a concomitant disappearing of main absorption band of the free 3′,4′-dihydroxyflavone.

If 3′,4′-dihydroxyflavone is in excess to copper ions (blue line), the spectrum shows only a small decrease in intensity of band at 340 nm, with the concomitant appearance of a low intensity flat band at 396 nm; band at 244 nm follows the spectrum of free 3′,4′-dihydroxyflavone.

In the UV-Vis spectrum of taxifolin, the absorption maximum is observed at 289 nm with a shoulder centered at around 326 nm (Figure 6). Inspection of the taxifolin structure indicates two potential binding sites for the cupric ions: the 4-CO/5-OH groups (A/C rings) and the 3′,4′-dihydroxy groups of the catechol moiety (B-ring) [31,32]. The presence of the saturated C2-C3 bond in the C-ring interrupts the delocalization of the π-system between the A/C fused rings and the B-ring which (unlike flavonols) become deconjugated and independent of one other. Consequently, the interaction of cupric ions through the 3-OH/4-CO groups is much less favourable, because the interaction is preferentially directed towards the formation of a more stable, conjugated planar system. UV-Vis spectra (Figure 6) recorded in the presence of cupric ions exhibit loss of the flat band at 326 nm and simultaneous appearance of a new, very low in intensity band at 390 nm. The observed spectral changes can be attributed to the weak Cu(II)-binding to the 3′,4′-dihydroxy groups of the catechol moiety (new band at 390 nm).

The UV-Vis spectrum of the coumarin derivative shows major peaks at 286 nm, 298 nm and ~345 nm (Figure 6, lower panel). However, following its interaction with cupric ions the first peak is slightly skewed to the lower wavelengths and the band at ~345 disappeared. Such negligible changes in the UV-Vis spectrum, following the interaction of coumarin with Cu(II) are in agreement with previous reports and can be explained by the isolated keto- or hydroxyl- groups in its molecular structure, which cannot strongly bind metal ions [31,33].

In summary, to effectively bind cupric ions to phenolic compounds requires the presence of the 3-OH and 4-CO groups of the flavonoid C ring, which is documented by the formation of a new band in the UV-Vis spectrum; however, adjacent -OH groups in ring B can also serve as donor groups to interact with cupric ions. The results further indicate that the tight interaction between phenolic compounds and cupric ions via the 3-OH and 4-CO groups is only achieved if the unsaturated C2-C3 bond of the C-ring is present, which permits through-conjugation with the B-ring. From a spectroscopic point of view, the more intense and more greatly shifted toward longer wavelengths is the newly-present band (>400 nm) in the UV-Vis spectra, the stronger is the interaction of cupric ions with the polyphenol, and this occurs preferentially through its 3-OH and 4-CO groups. Coordination of the copper(II) ions to the 3′-OH and 4′-OH groups of the polyphenol [33] is signified in the UV-Vis spectra by the appearance of a less intense band at wavelengths greater than 400 nm. According to the UV-Vis data, the phenolic compound most tightly coordinated to Cu(II) ions, within the series of compounds studied is myricetin and morin.

### 2.2. Antioxidant Properties of Phenolic Compounds and Their Cu(II) Complexes

The antioxidant (radical scavenging) activity of phenolic compounds and their complexes with cupric ions (prepared in situ using metal-to-ligand ratios of 1:1 and 1:2) was monitored using the ABTS assay, which, briefly, is a simple non-enzymatic method commonly used for the in vitro evaluation of the antioxidant activity of H-donor substances. Formation of the blue-green coloured ABTS radical cations (ABTS^•+^) is initiated chemically and their decay, following addition of the compound being studied is monitored using UV-Vis spectroscopic measurements of the ABTS^•+^ absorption band at 734 nm. The reaction between ABTS^•+^ and the phenolic compound is accompanied by the decolorization of the ABTS^•+^ as it is reduced back to its colourless neutral form. Radical scavenging activity is one of the criteria that a substance must meet to be described as an antioxidant, and so those molecules with better radical scavenging activity are generally considered to have better antioxidant properties. One should bear in mind, however, that the antioxidant activities of phenolic compounds also indirectly depend on their metal-chelating abilities, as discussed above.

The results of the ABTS assay are summarized in Table 1, which clearly show there are significant differences in radical scavenging activities among the individual phenolic compounds.

The observed differences in radical scavenging activities of phenolic compounds are related to their molecular structures and decrease in the order: MYR > MOR > DHF ~ CUM > TAX. Generally, there are two main mechanisms through which phenolic compounds can exert their scavenging activity: the hydrogen transfer mechanism and electron donation mechanism. In the ABTS assay, the ABTS^•+^ reacts with hydrogen donor molecules and therefore we can expect a positive correlation between the radical scavenging activity of phenolic compounds and the number of their hydroxyl groups [34]. Myricetin, with six hydroxyl groups, exerts the highest antioxidant activity, followed by morin which possesses five hydroxyl groups. In the presence of Cu(II) ions, the antioxidant activity of both myricetin and morin decreases because the coordinated cupric ions replace hydrogen atoms and thus the ability of phenolic molecule to provide hydrogen atoms for reaction with ABTS radical is reduced.

3′,4′-Dihydroxyflavone with two hydroxyl groups achieves about 50% of the scavenging activity of morin. A common structural characteristic of myricetin, morin and 3′,4′-dihydroxyflavone is the presence of the planar 2-phenyl-benzo-γ-pyrone skeleton. A significantly lower activity of the pure flavone compounds, as compared with the flavonols, correlates with their smaller number of hydroxyl substituents.

Although the molecule of 3′,4′-dihydroxyflavone has only one site for copper chelation (3′-OH and 4′-OH), the radical scavenging activity is about the same for the pure flavone, or the flavone in the presence of cupric ions in either molar ratio, which can be attributed to a weaker chelation ability observed in fresh prepared solution (after 10 min), as is indicated by the UV-Vis spectrum of this flavone (Figure 5). The higher scavenging activity of the 3′,4′-dihydroxyflavone, (with just two hydroxyl groups) compared to that of taxifolin (which has five hydroxyl groups), can be explained in terms of the radical stability associated with the above mentioned, planar, 2-phenyl-benzo-γ-pyrone skeleton. While the entire 3′,4′-dihydroxyflavone molecule possesses an entire conjugated system, in which the radical is extensively stabilized by resonance, in the taxifolin molecule, the conjugation of the system is disrupted by the absence of a C2-C3 double bond and the resulting radical will be, accordingly, less stable.

The antioxidant activity of 4-hydroxycoumarin is comparable with 3′,4′-dihydroxyflavone (one hydroxyl group vs. two hydroxyl groups); however, the separation between the hydroxyl and carbonyl groups of 4-hydroxycoumarin does not allow them to provide a site for copper chelation (as documented by the UV-Vis spectra) and thus the activity of 4-hydroxycoumarin in the presence of Cu(II) ions is almost identical to the free compound.

In conclusion, when overviewing the reasons for the observed differences in antioxidant/radical scavenging activities of the phenolic compounds studied, there are three main factors that must be considered: (i) the number of hydroxyl groups (i.e., the number of potentially donatable hydrogen atoms) (ii) the overall planarity of the molecular skeleton and (iii) the extent of the electronically delocalized system. Molecules with more extended conjugated systems form resonance-stabilized free radicals, which have more markedly increased half-lives.

It should be noted that the ABTS assay experiments express the relative rate of antioxidant activity, as determined under in vitro conditions, which is significantly different from the physiological state in the cells and biological fluids of living organisms [35]. In order to draw conclusions that are most relevant to living systems, a more comprehensive assessment under different experimental conditions is needed.

### 2.3. DNA Interaction Studies Using Absorption Titrations

Electronic absorption spectroscopy is a sensitive method for determining the binding characteristics of small organic molecules and/or their metal complexes with a DNA molecule. Changes observed in the electronic absorption spectrum reflect the interaction between the molecules of a particular compound/complex and DNA, and inferences may be drawn regarding the mechanism of this interaction from wavelength shifts of the corresponding absorption bands. Furthermore, by determining the internal binding constant K_b_, a more quantitative basis may be established for the process [36,37,38,39].

In summary, we recall that small organic molecules and transition metal complexes can interact with DNA either by covalent bonding and/or non-covalent interactions, for example by (i) intercalation into DNA, (ii) electrostatic interactions involving phosphate groups that are present on the DNA chains and (iii) interaction with major or minor grooves in the DNA [40]. The mechanism of interaction depends on the structure of the molecule and thus, planar molecules with conjugated bonds interact with DNA most frequently by intercalation, so creating “stacking” interactions of the aromatic chromophore with DNA bases [41]. Generally, if the molecule interacts with DNA via intercalation, both hypochromism and bathochromism (red shift) are observed in the absorption spectrum. Bathochromism can be explained on the basis of the coupling of an antibonding π* orbital of the intercalating ligand and the π-orbital of the DNA base pairs, which thus lowers the π → π* transition energy, and in turn results in a shift to longer wavelengths. Given that the coupled π orbital is not fully filled with electrons, there is a decrease in the values of the spectral transition probabilities and this is reflected in the spectrum as a decrease in absorbance (hypochromism) [42,43,44]. Thus, the binding of a molecule to DNA via intercalation involves a stacking interaction between the aromatic moiety of the molecule and the base pairs of DNA, which is characterized by both a hypochromic shift and a red shift (bathochromism) in the electronic absorption spectra. The strength of binding of the complexes with DNA is determined quantitatively by evaluating the binding constant K_b_. Electrostatic interactions or hydrogen bonding between the substance and DNA result in destabilization of the latter, which is manifested in the spectrum by a hyperchromic shift [45].

In our experiments, we investigated the effect of adding of CT-DNA to a solution of phenolic compounds or copper-phenolic complexes (Cu:phenolic compound = 1:2). Electronic transitions at characteristic wavelengths can be observed in the absorption spectra of organic compounds, whose molecules exhibit σ-σ* transitions in the range of 150–250 nm; π-π* transitions in systems with conjugated bonds typically occur in the range of 200–400 nm; while n-π* transitions (with the lowest energy of all) are observed at wavelengths longer than 400 nm (400–700 nm).

**Figure 7:**
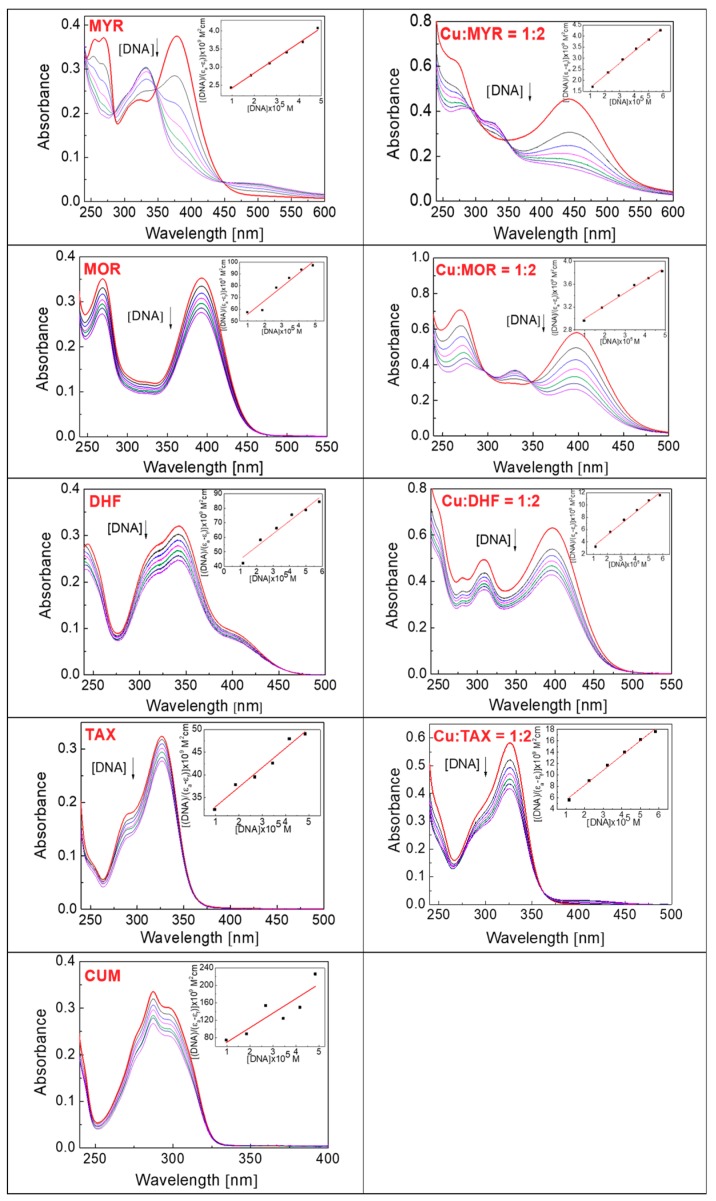
Absorption spectra obtained by titration of free phenolic compound (20 μM) and phenolic compound in the presence of Cu(II) (Cu:phenolic compound = 1:2) with CT-DNA (0–50 μM) in a solution containing 5 mM Tris-HCl, 50 mM NaCl, pH 7.2 in the absence of CT-DNA (red line) and in the presence of an increasing concentration of CT-DNA (0–50 μM). The direction of the arrow indicates the shift in the absorbance after addition of the CT-DNA solution. Abbreviations: myricetin (MYR), morin (MOR), 3′,4′-dihydroxyflavone (DHF), taxifolin (TAX), 4-hydroxycoumarin (CUM).

Figure 7 shows the absorption spectra of myricetin, morin, 3′,4′-dihydroxyflavone, taxifolin and 4-hydroxycoumarin (both as the free phenolic compounds and as their Cu:phenolic (1:2) chelates) after addition of an aliquot of CT-DNA solution: the occurrence of spectral shifts observed for flavonoids in the presence of cupric ions relative to free flavonoids confirms the existence of Cu(II)-flavonoid chelates in solution.

Please note that slight shifts in the absorption maxima of free phenolic compounds observed in the DNA interaction studies (this Section) and UV-Vis spectroscopy study (Section 2.1.2) are caused by the different compositions of the buffers used.

The electronic absorption spectrum of free myricetin (MYR) is more complex than that of the other phenolic compounds, with significantly intense peaks at 267 nm and 378 nm. It was found that the intensities of both peaks decreased as the concentration of added DNA increased (Figure 7, Table 2), and for the band at 267 nm, a hypochromic shift of about 45.95% was observed, accompanied by a small bathochromic shift (of about 8 nm).

The band at 378 nm was found to decrease in intensity by up to 72.97% with a hypsochromic shift (of about 5 nm). The myricetin spectrum also contains a less intense peak at 322 nm, but which gains intensity as the concentration of DNA increases showing a hyperchromic shift (of about 23.33%) and also exhibits a bathochromic shift (of about 10 nm). There are three isosbestic points in the spectrum at 284 nm, 347 nm and 447 nm, which indicate the existence of different binding mechanisms of MYR to DNA. The binding constants of the phenolic compounds and their Cu(II) complexes with DNA are measures of the strength of the interaction and are summarized in Table 3; the calculated value of binding constant for the interaction of free myricetin with DNA is 2.07 × 10^4^.

The electronic absorption spectrum of the Cu:MYR (1:2) complex has features similar to those for free myricetin, with the exception that the band at 267 nm is flat and poorly resolved; the band at 441 nm is more intense and rather broad. Both bands show a hypochromic shift with increased DNA concentration: for the band centered at 267 nm the decrease in intensity is 36.51% and is associated with a hypsochromic shift of about 5 nm; the intensity of the band at 441 nm is reduced by 66.67%, and is accompanied by a small bathochromic shift of about 2 nm. Similarly to free myricetin, addition of DNA to the solution of the Cu:MYR (1:2) complex results in hyperchromic shift of the band at 346 nm (23.33%) accompanied by a hypsochromic shift (about 21 nm); the isosbestic points are at 292 nm and 350 nm. The calculated value of the binding constant for the interaction between the Cu-MYR (1:2) complex and DNA is 4.02 × 10^4^, which indicates a slightly higher strength of the intercalation process than occurs for free myricetin (Table 3). The observed hypochromism confirms that intercalation has taken place between myricetin, and its Cu-complex and DNA. In addition, the observations of isosbestic points and hyperchromism indicate that, in addition to intercalation, other mechanisms of interaction, such as electrostatic and covalent interactions have occurred. Disruption of the secondary DNA structure may occur, as a result of covalent interactions between the Cu-MYR complex and the negatively charged DNA phosphate backbone with subsequent cleavage.

The absorption spectrum of free morin (MOR) has two major bands at 269 nm and 393 nm, and the gradual addition of CT-DNA into a solution containing morin resulted in the hypochromic shifts of both bands: approximately 22.86% at 269 nm and 21.64% at 393 nm (Table 2). Based on this observed hypochromism, we can assume that an intercalation mechanism occurs between free morin and DNA, most probably mediated by the benzoyl moiety as is confirmed by the estimated value of the binding constant, 3.23 × 10^4^.

Similar hypochromic shifts as that observed for the Cu-MYR (1:2) complex were also noted for the Cu-MOR (1:2) system after the addition of CT-DNA. Two absorption maxima, one centered at 269 nm and another at 398 nm, exhibited pronounced hypochromic shifts of 42.25% and 55.17%, respectively. The band at 269 nm also showed a significant bathochromic shift of about 7 nm, while an almost negligible hypsochromic (blue) shift (~2 nm) was observed for the 398 nm band; isosbestic points were observed at 297 nm and 348 nm. The value of DNA binding constant for Cu-MOR (1:2) complex was found to be 8.08 × 10^3^, which suggests that this complex intercalates less tightly into the DNA than was observed either for free morin (3.23 × 10^4^) or for MYR and the Cu-MYR (1:2) complex (Table 3). The existence of a hyperchromic shift in the UV-Vis spectrum suggests that other types of interactions, for example of electrostatic and covalent kinds, also occurred between the Cu-complex and DNA.

The spectroscopic data discussed above indicate that the interaction of free morin or myricetin and their Cu-complexes with DNA is more than a simple intercalation process, and that other types of interaction with the DNA molecule also occur. While hypochromism, accompanied by batochromism, is typically attributed to intercalation mechanisms, the hyperchromic effect may be the result of electrostatic interactions between the positively charged flavonoid molecule, or its copper chelate, with the negatively charged phosphate groups of DNA.

3′,4′-Dihydroxyflavone (DHF) exhibits absorption maxima at 244 nm and 342 nm (Figure 7) and following the addition of the CT-DNA solution, a hypochromic shift in the spectrum occurred, of approximately 17.86% and 22.83%, as is indicated for the corresponding peaks (Table 2). The spectrum recorded from the system containing 3′,4′-dihydroxyflavone and Cu(II) ions (Cu-DHF = 1:2) shows two intense peaks at 308 nm and 397 nm and a lower intensity peak at 281 nm. After addition of DNA, hypochromic shifts were measured of 26.83% (at 281 nm), 28% (at 308 nm) and 31.75% (at 397 nm) (Table 2). The absorption spectra of both dihydroxyflavone and its copper(II) chelates show a hypochromic shift (no bathochromic shift was observed) which identifies that the intercalation mechanism with DNA had occurred. In principle, hypochromism without any significant shift to longer wavelengths (bathochromism) might be due to the loss of chromophore molecules into the solution that have been taken up by the DNA molecule during the course of the titration [42]. From a consideration of the molecular structure of 3′,4′-dihydroxyflavone, it seems probable that the molecule will intercalate into DNA via its benzoyl moiety. The binding constants determined for the interaction of free 3′,4′-dihydroxyflavone and the Cu-DHF (1:2) complex with DNA are 2.43 × 10^4^ and 1.23 × 10^5^, respectively (Table 3).

While the hypochromism and value of the binding constant for free dihydroxyflavone confirms its interaction with DNA by the intercalation mechanism (~10^4^), the binding constant of the Cu-DHF complex with DNA is higher by one order of magnitude (~10^5^) which confirms that the interaction between this complex and DNA is much stronger.

The absorption spectrum obtained by titrating a solution of free taxifolin (TAX) and taxifolin in the presence of cupric ions (Cu:TAX = 1:2) with DNA is shown in Figure 7, which shows an intense absorption band at 326 nm, whose intensity is reduced by titration with CT-DNA solution. This hypochromic shift (25.37%) is the result of the interaction of taxifolin with DNA by the intercalation mechanism (Table 2); a binding constant value of 1.44 × 10^4^ was determined which confirms the function of a mild intercalation mechanism (Table 3). The absorption spectrum of the complex Cu-TAX (1:2) has a maximum at the same wavelength as the free taxifolin, indicating that copper(II) is chelated by taxifolin either only very weakly or not at all. The observed hypochromic shift (27.59%) is the result of the interaction of taxifolin, and not the Cu-TAX (1:2) complex with CT-DNA; a weak copper chelating abilities of taxifolin are also evident from the UV-Vis spectra (Figure 6).

As indicated above, the UV-Vis spectra (Figure 6) demonstrate that 4-hydroxycoumarin does not interact with cupric ions, and hence electronic absorption titrations were performed only for the free phenolic compound. The effect of adding an aliquot of CT-DNA solution to a constant concentration of free 4-hydroxycoumarin is shown in Figure 7, the spectrum showing an intense maximum at 287 nm. The hypochromic shift is evident from the spectrum (20.59% at 287 nm) and indicates the interaction of coumarin with the DNA by weak intercalation, for which the binding constant is 6.61 × 10^4^ (Table 3).

According to their binding constant values, we may order the free phenolic substances in order of decreasing binding strength as follows: CUM > MOR ~ DHF ~ MYR > TAX, and very interestingly, the highest interaction strength was observed for the smallest molecule, 4-hydroxycoumarin. For systems containing copper ions, we noted an increase in the binding constant for Cu(II)-dihydroxyflavone, and for Cu(II)-myricetin complexes over that for the free phenolic compounds, which indicates a potentially important role of cupric ions as a transport vehicle for their intercalation into DNA. Conversely, for the Cu-MOR complex, a decreased strength of interaction with DNA was observed, as compared with that for free morin.

From the above, we can conclude that myricetin can interact with Cu(II) via donor atoms in the C ring (3-OH and 4-CO groups) and the B ring (3′-OH and 4′-OH groups) and that it exhibits an interaction with DNA of intermediate strength (K_b_ ~ 10^4^); morin, which interacts exclusively via donor atoms on the C ring (3-OH and 4-CO groups) exhibits a mild interaction strength with DNA (K_b_ ~ 10^3^), while 3′,4′-dihydroxyflavone, which can interact exclusively via the 3′-OH and 4′-OH groups on the ring B, exhibits a strong interaction with DNA (K_b_ ~ 10^5^). The overall results indicate that copper ions can modulate the DNA binding affinity of flavonoids via the formation of their Cu-chelates.

Mechanisms by which myricetin, morin and 3′,4′-dihydroxyflavone and their Cu(II) complexes can interact with DNA predispose these substances to act as potential anticancer agents. Weak prooxidant properties of phenolic compounds under study make them promising substances to boost cellular antioxidant systems including antioxidant enzymes and low molecular antioxidants such as glutathione.

### 2.4. Protective Effect of Phenolic Compounds against DNA Damage

DNA damage is associated with several diseases, as well as premature aging. Protection of DNA from damage is important because of the important role of this molecule in the transfer of genetic information and cell division. It is believed that phenolic compounds are suitable/promising natural antioxidants that could prevent unwanted damage to DNA and in order to evaluate their potential in this respect, gel electrophoresis was used with plasmid DNA (pDNA) under both normal and Fenton conditions.

Gel electrophoresis represents a relatively simple way to evaluate radical induced DNA damage (in the presence of Fenton like agents) and/or damage caused by intercalating molecules. If oxidative cleavage to the covalent DNA bonds occurs, new conformations of pDNA can arise: cleavage in one strand of the native superspiralized (SC) form of pDNA results in an open circular conformation (OC), while cleavage in both strands results in a linear form (LIN). Both these DNA conformations differ in their mobility in the gel and are made visible by staining with ethidium bromide [46]. From the band intensities of the DNA conformational states, their relative abundances may be determined. In our experiments, the reaction mixture contained cupric ions and a phenolic compound (with a constant copper concentration and an increasing concentration of the phenolic compound). Experiments were performed both in the absence, and presence of hydrogen peroxide (Fenton reaction (1)). The addition of hydrogen peroxide (50 μM) followed by the DNA addition initiates the reaction with the copper ions and the subsequent Cu(II)/Cu(I) redox cycle, which results in the formation of ROS. The degree of DNA damage was correlated with the antioxidant (suppression of DNA damage) or prooxidant (increase of DNA damage) effect of the given phenolic compound.

A protective/antioxidant effect of phenolic compounds (especially polyphenols) is generally believed to be due to a combination of: (i) their metal (e.g., copper) chelating properties, resulting in suppressed catalytic activity of the metal ions in the Fenton reaction, and/or ii) a direct scavenging of free radicals [47]. In other words, the copper chelation by flavonoids reduces the ability of Cu(II) ions to enter the redox cycling mechanism, initiated by hydrogen peroxide, which would otherwise result in DNA damage. The protective effect of copper chelation by flavonoids is enhanced when the Cu-flavonoid complex is formed before hydrogen peroxide has been added. Generally, an increase in the intensity of the SC form of pDNA (preserved SC DNA) with a concomitant decrease of the OC and/or LIN form intensities, when the phenolic compound is present, is considered to signify that the substance being studied has exerted a protective effect. Generally, it is expected that increasing the concentration of phenolic compounds enhances the DNA protective effect predominantly via a radical scavenging mechanism.

**Figure 8:**
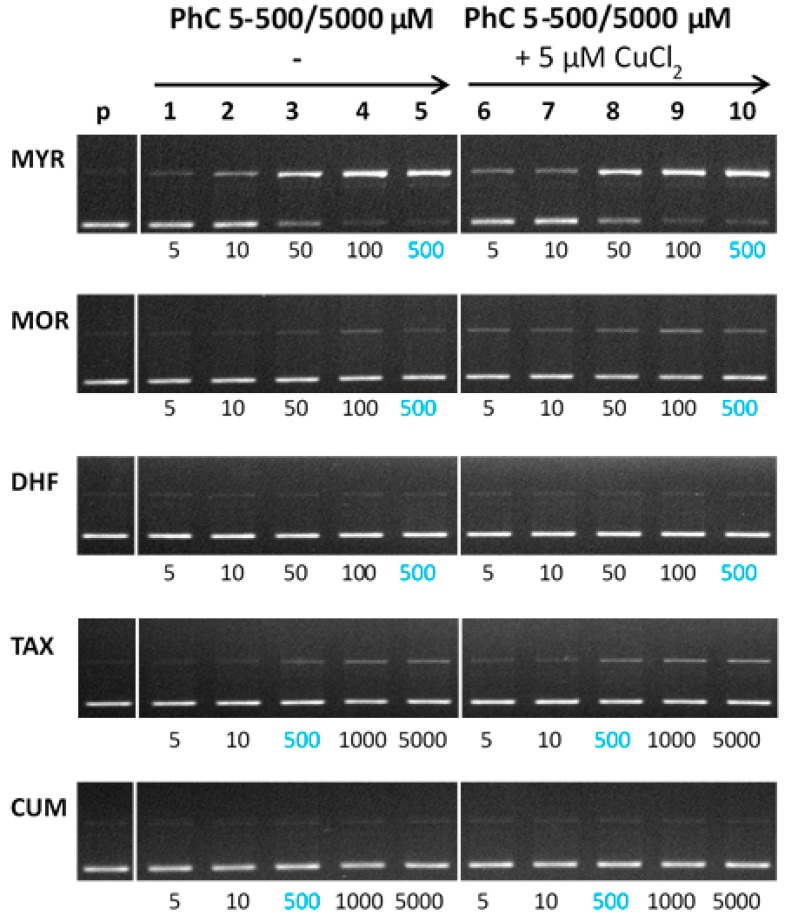
Gel electrophoregram of the interaction of phenolic compounds (PhC) and their Cu(II) complexes with plasmid DNA in the absence of hydrogen peroxide (non-Fenton system). Electrophoretic profile of agarose gel (0.8%) of the reaction mixture of 20 µM pBSK+ DNA, free phenolic compounds (lanes 1–5) and Cu(II) complexes with phenolic compounds (lanes 6–10), incubated at RT for 30 min. The order of the columns in the gel: (p) control pDNA; (1–5) pDNA + 5–500/5000 µM PhC (MYR, MOR, DHF/TAX, CUM), (6–10) pDNA + 5–500/5000 µM PhC (MYR, MOR, DHF/TAX, CUM) + 5 µM Cu(II). Experimental conditions of all reactions were the same, so the observed differences in gels are related to structural differences of phenolic compounds. Due to the different solubility of phenolic compounds, individual phenolic compounds were used under different concentrations. (PhC = Phenolic compound; MYR = Myricetin; MOR = Morin; DHF = 3′,4′-dihydroxyflavone; TAX = Taxifolin; CUM = 4-hydroxycoumarin).

Figure 8 shows the electrophoregram of the interaction of free phenolic compounds and their Cu(II)-complexes with plasmid DNA in the absence of hydrogen peroxide (non-Fenton experiment). In the absence of Cu(II) (see left panel, Figure 8), the most profound concentration dependent DNA damage is noted for myricetin. A slight degree of DNA damage has also been observed for the interaction of taxifolin with DNA. A similar trend as it was observed for free phenolic compounds interacting with DNA was observed for their Cu(II) complexes.

Interestingly, the Cu-MYR complex exhibit similar degree of DNA damage as it was observed for free myricetin, which roughly correlates with the similar binding constants for MYR and the Cu-MYR complex with DNA (~10^4^). From the electrophoretic profiles obtained, it may be deduced that the most severe DNA damage is caused by the myricetin, which contains a 3-OH group on ring C and three adjacent -OH groups on ring B. Through these functional groups, myricetin can interact with cupric ions. A weakly damaging effect of taxifolin and its Cu(II) complex accords with the presence of a 3-OH group on ring C and two adjacent -OH groups on ring B; however, the C2-C3 bond of ring C is saturated, which interrupts the extent of conjugation and coplanarity that are important for the interaction with cupric ions. Coplanarity of the molecule is also important for the intercalation mechanism of action with DNA.

**Figure 9:**
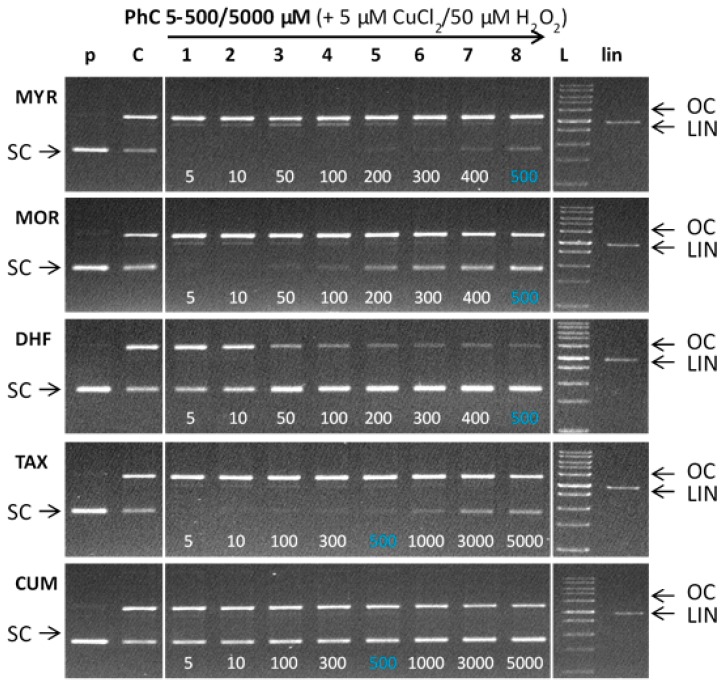
Gel electrophoregram of the interaction of phenolic compounds (PhC) and plasmid DNA in the presence of hydrogen peroxide (Fenton system). Electrophoretic profile (0.8% agarose gel) of DNA protection against oxidative damage in the reaction mixture (20 µL) containing Cu(II) ions, phenolic compounds, pBSK+ DNA, hydrogen peroxide and incubated at RT for 30 min. The order of the samples in the gel: (p) control pDNA; (C) Fenton like reaction containing pDNA + 5 µM CuCl_2_ + 50 µM H_2_O_2_; (1–8) Fenton like reaction + phenolic compound at concentrations of 5–500 µM (MYR, MOR, DHF) or 5–5000 µM (TAX, CUM); (L) 1 kb DNA standard (LIN) linearized pDNA.

Figure 9 shows the gel electrophoregram of the interaction of phenolic compounds (PhC) and their Cu(II) complexes with plasmid DNA in the presence of hydrogen peroxide (Fenton-like system). Generally, damage observed for a Fenton-like system is greater than for a non-Fenton system, due to the ROS formed in the course of the Fenton reaction. Similarly to the non-Fenton system, the studied flavonoids, myricetin, morin, and 3′,4′-dihydroxyflavone were tested within the concentration range 5–500 μM, and the flavonoids TAX and CUM allowed their testing up to the 5000 μM concentrations (lanes 1–8). Each electrophoretic profile contains a control pDNA (lane p) indicating the quality of the pDNA used in each reaction and control reaction with Fenton agents (cupric ions, H_2_O_2_) (lane C) showing new conformational states of pDNA in the absence of phenolic compounds. Thus the lane C shows the DNA damage caused exclusively by ROS formed in the course of the copper-catalyzed Fenton reaction (of which the main ROS is the hydroxyl radical).

As can be seen from the electrophoretic profiles, the flavonoids myricetin, morin, and taxifolin show pro-oxidant behaviour being documented in gels by the complete loss or significant suppression of bands corresponding to SC pDNA (Figure 9, MYR—lanes 1–5; MOR—lanes 1–3; —lanes 1–2) accompanied by the enhanced band intensities corresponding to the cleaved plasmid DNA (OC and LIN). In cases of morin and taxifolin and also partly in the case of myricetin the DNA damage can be accounted for by ROS products originating from Fenton reaction.

A weak protective effect of myricetin was observed at a concentration of 400 μM, which is well above the copper concentration (Figure 9, MYR—lane 7), but in comparison with the control variant (lane C), the loss of SC DNA is still significantly greater because of the interaction of Cu-myricetin complex with DNA (see Figure 8). Even at the highest concentration tested (500 µM, lane 8), myricetin did not achieve a protective effect comparable to the effect of morin at 200 μM concentration (MOR—lane 5), in agreement with the non-Fenton experiments (see Figure 8). In the case of morin, a gradual increase in the band intensity of the SC form over a concentration range of 100–500 µM (MOR, lanes 4–8) suggests a stronger protective effect, as compared with myricetin. Considering the similarity in the structures of myricetin and morin (differing by just one -OH group), this finding is rather unexpected, because the protective effect of flavonoids (or generally antioxidants) against ROS is usually related to their number of hydroxyl substituents.

Thus, the strong prooxidant behaviour of myricetin under the conditions of the Cu-Fenton reaction must be related to a different mechanism of action. We propose that the formation of dimeric or even polymeric species of Cu:myricetin complex, as documented by the EPR spectroscopic measurements (see above) and hyperchromism observed in the absorption titrations (see above), point to the complex mechanism of action of the Cu(myricetin)_2_ complex causing significant DNA damage. In addition, dimeric/polymeric Cu complexes with chelated myricetin have a less tightly saturated coordination sphere around the copper ion than do monomeric Cu complexes, so the catalytic activity of the Cu-complex in the Fenton reaction is more profound and results in the formation of more ROS. The prooxidant behaviour of myricetin in the presence of cupric ions indicates its possible use as an anticancer agent [48]. From the results obtained, it follows that the DNA protective effect of flavonoids against ROS, in the presence of redox metals such as copper, may not necessarily be related to the number of hydroxyl groups in the molecular structure of the flavonoid. The presence of 3-OH group on ring C, which is involved in the interaction with cupric ions, and planarity of the flavonoid, are important factors that contribute to its prooxidant activity, and also the presence of adjacent -OH groups on ring B, which can also assist in its interaction with cupric ions. Thus, the combined interaction via donor atoms on ring C and ring B results in the formation of stable Cu-flavonoid complexes that are capable of causing severe DNA damage. Thus, to have a high number of hydroxyl groups, which otherwise is a beneficial structural feature of flavonoids, may become counterproductive in the presence of metal ions (e.g., Cu^2+^), especially when a 3-OH group is present on ring C, and there are adjacent -OH groups on ring B which can all participate in the interaction between the flavonoid and cupric ions.

As illustrated in Figure 9, taxifolin shows a weaker, but similar, trend to myricetin. The presence of a weak band from the SC form at a concentration of 100 µM (TAX—lane 3) indicates a somewhat weaker pro-oxidant effect as compared with the same concentration of myricetin (MYR—lane 4). However, a certain level of prooxidant activity for taxifolin is observed even at its highest (5000 µM) concentration (TAX—lane 8). The prooxidant effect of taxifolin, in the presence of cupric ions, can be explained on the basis of ROS-induced damage and to some extent by DNA intercalation mechanism of action (Figure 8). Taxifolin also shows a strong tendency to form dimeric species when coordinated to Cu(II) ions.

In contrast, 3′, 4′-dihydroxyflavone, significantly, and 4-hydroxycoumarin (no 3-OH group), to some extent, both exhibit a protective effect within the entire concentration range tested, as is illustrated by the degree of preservation of the SC form of plasmid DNA in gels (Figure 9, DHF—lanes 1–8, CUM—lanes 1–8). A more pronounced concentration dependence is observed in the presence of DHF, as seen in the gel by a gradual significant loss of OC pDNA. A very weak prooxidant effect of dihydroxyflavone can be explained on the basis of its ROS-scavenging activity and by the existence of only one potential copper chelating site (two hydroxyl groups on ring B) so creating a more labile copper complex that cannot cause significant damage to DNA.

4-Hydroxycoumarin has no potential site to chelate cupric ions and therefore any potential DNA damage caused by intercalation of its copper complex can be excluded (see also Figure 8). The overall damage observed is due to the Cu-catalyzed Fenton reaction suppressed by the radical scavenging activity of free (uncoordinated) 4-hydroxycoumarin.

**Figure 10:**
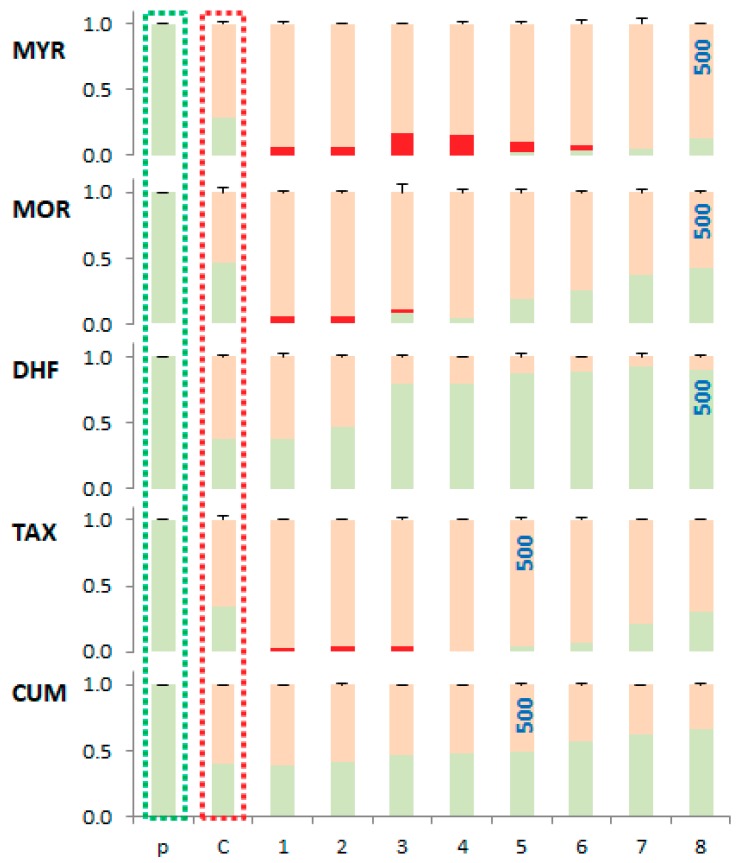
Relative densitometric quantification of band intensities of, at least, 3 agarose gels as illustrated in Figure 9. Green colour corresponds to the SC form of plasmid DNA, red colour to the LIN form and orange colour to the OC form. Taxifolin and coumarin were tested up to a concentration of 5000 μM. p—SC form of pDNA, **C**—proportion of pDNA conformations formed in Fenton like reaction, 1–8—proportion of pDNA conformations formed in Fenton like reaction in the presence of phenolic compound. Standard deviations are expressed as error bars.

Quantification of band intensities was performed by densitometric analysis. The relative concentration proportions of the individual conformational forms of plasmid DNA are shown in Figure 10.

Based on the electrophoretic profiles, the protective effect of the phenolic compounds studied, against plasmid DNA damage under the experimental conditions described, decreases in the order: DHF > CUM > MOR > TAX ~ MYR. The results clearly show that more pronounced DNA damage occurs in the presence of those flavonoids with more hydroxyl groups such as MYR (with 6-OH groups, and where a 3-OH group is present), MOR (with 5-OH groups, and a 3-OH group is present) and TAX (with 5-OH groups and where a 3-OH group is present) all form stable copper(II) complexes, that are capable of causing DNA damage via interaction with DNA and ROS formation via Fenton reaction [49].

### 2.5. Qualitative Analysis of ROS Formation

During the course of the Fenton reaction, ROS such as singlet oxygen (^1^O_2_), hydroxyl radical (^•^OH), and superoxide anion radical (O_2_^•−^) are formed. In order to identify these species, the following radical scavengers, L-histidine (detection of singlet oxygen), DMSO (detection of hydroxyl radical) and SOD enzyme (detection of superoxide radical) were incorporated into the reaction system.

**Figure 11:**
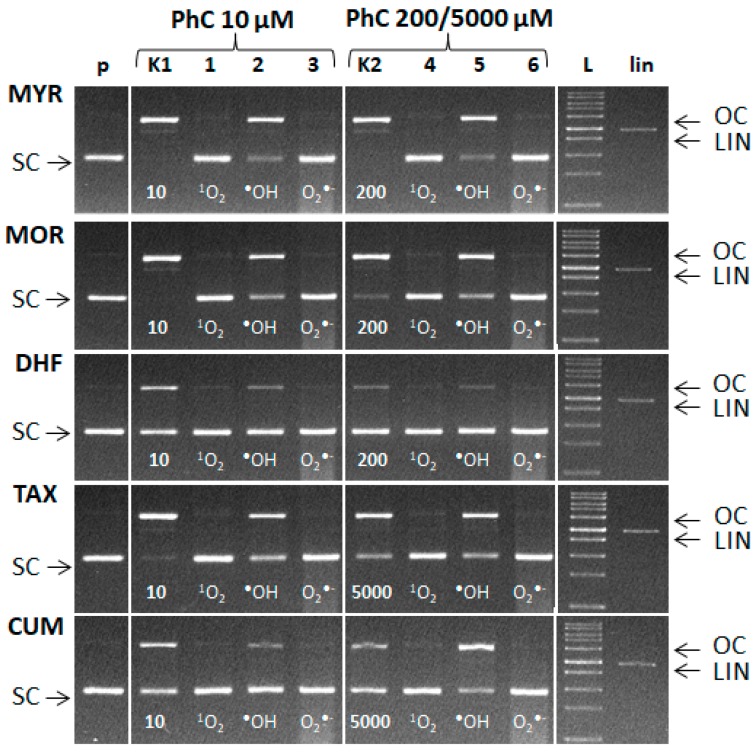
Evidence of ROS formation in the Fenton-like system. Electrophoretic profile of agarose gel (0.8%) of the reaction mixture of 20 µM pBSK+, Cu(II) ions, hydrogen peroxide and phenolic compounds incubated at room temperature for 30 min. The order of the samples in the gel: (p) control pDNA; (K1) Fenton like reaction containing pDNA + 5 µM CuCl_2_ + 50 µM H_2_O_2_ and 10 µM of phenolic compound; lanes 1–3: Fenton like -reaction and 10 µM of phenolic compounds in the presence of l-Histidine (lane 1), DMSO (lane 2) and SOD (lane 3); (K2) Fenton-like reaction and 200 µM of MYR, MOR, DHF or 5000 µM of TAX and CUM. lanes 4–6: Fenton like reaction and 200 µM of MYR, MOR, DHF or 5000 µM of TAX, CUM in the presence of l-Histidine (lane 4), DMSO (lane 5) and SOD (lane 6). ROS scavengers were added in following amounts: l-Histidine 20 mM, DMSO 6 μL, SOD 15U.

Since the amount of ROS formed is not known, scavengers were applied in a relatively high concentration. The experiments were carried out using two stoichiometric molar ratios of Cu: phenolic compound. The molar ratio Cu(II):PhC = 1:2 was used (Figure 11—lanes K1 and 1–3). In a second variant the phenolic compound was in a large excess (Cu:MYR/MOR/DHF = 1:40; Cu:TAX/CUM = 1:1000) (Figure 11—lanes K2 and 4–6).

Figure 11 documents the formation of singlet oxygen, hydroxyl radicals and superoxide radical anions in the course of the Cu-Fenton reaction in the presence of plasmid DNA. From the observed band intensities we can conclude that the addition of L-histidine (scavenger of ^1^O_2_) or SOD (scavenger of O_2_^•−^) significantly preserved SC DNA. Interestingly, addition of DMSO (scavenger of ^•^OH) only partially preserved SC DNA (see rather intense bands of OC DNA). This can be explained by the transformation of hydroxyl radical to the methyl radical (^•^CH_3_) which can cause DNA damage to some extent, as a secondary radical, according to the reaction [50]:

(CH_3_)SO + ^•^OH → ^•^CH_3_ + CH_3_SO_2_H (2)

It may be speculated that the preservation of SC DNA after addition of either L-histidine or SOD (scavengers of superoxide radical or singlet oxygen, respectively) to the reaction system is achieved by the scavenging of additional ROS generated in the system at the expense of the scavenging activity of a given phenolic compound.

From the electrophoretic profiles also follows that scavenging of one of the formed ROS results in significant preservation of SC DNA. This points to fact, that concerted action of ROS of various origin has synergistic damaging effect on DNA damage [51].

## 3. Materials and Methods

### 3.1. Materials

Phenolic compounds myricetin (MYR), taxifolin (TAX), 4-hydroxycoumarin (CUM) were purchased from Sigma Aldrich (Steinheim, Germany), 3′,4′-dihydroxyflavone (DHF) and morin (MOR) were obtained from Alfa Aesar (Ward Hill, MA USA). Myricetin, morin and 3′,4′-dihydroxyflavone contain a flavone structure (2-phenylbenzo-γ-pyrone) in which, because of presence of a double bond between carbons 2 and 3 of C ring, all three rings form a planar delocalized system. The molecule of 3′,4′-dihydroxyflavone contains a 3′,4′-dihydroxyphenyl group on the B-ring (catechol moiety). Myricetin and morin belong to the class of flavonols, and their structures represent isomeric forms of quercetin having a resorcinol moiety (A-ring) and a 3-hydroxy-4-carbonyl group on the C-ring, but they differ in the hydroxylation pattern of the B-ring. While myricetin possesses a pyrogallol moiety (3′,4′,5′-trihydroxyl), morin has two hydroxyl groups in a *meta*-2′,4′-position. The presence of a 4-carbonyl group on the C-ring and the hydroxylation pattern of taxifolin also corresponds with the quercetin structure, but the lack of a C2-C3 double bond disrupts planarity and conjugation of the rings. The 4-hydroxycoumarin is a coumarin (*2H*-chromen-2-one) derivative and represents the simplest molecular structure included in the study.

Other chemicals and reagents were procured from different commercial companies: disodium salt of calf thymus DNA (CT DNA), NaH_2_PO_4_, Na_2_HPO_4_, CuCl_2_·2H_2_O (cupric chloride dihydrate), H_2_O_2_ (30%), DMSO, DPPH (diphenyl-picrylhydrazyl), ABTS (2,2′-azino-bis(3-ethylbenzothiazoline-6-sulphonic acid) from Sigma Aldrich, absolute ethanol, TRIS base, agarose, ethidium bromide from Serva (Heidelberg, Germany), glycerol from Merck (Darmstadt, Germany), the 10×TBE (Trisborate-EDTA) and bromphenol blue from Applichem (Darmstadt, Germany). The 1 kb DNA ladder was purchased from Solis BioDyne (Tartu, Estonia) and *EcoRI* restriction enzyme from Thermo Fisher Scientific (Vienna, Austria). All chemicals were of analytical grade purity and used without any purification. The solutions used in DNA experiments were prepared using purified water (Simplicity Ultrapure Water System, Millipore, Burlington, MA, USA).

### 3.2. Experimental Solutions

Solutions of phenolic compounds: Stock solutions of phenolic compounds were prepared in DMSO (3 mM) for both spectroscopic studies and DNA experiments. The solutions were always prepared fresh and kept in the dark to avoid possible light induced changes.

Solution of CT DNA: Sodium CT DNA salt was gradually dissolved under continuous stirring in Tris buffer Saline (TBS) solution (5 mM TRIS, 50 mM NaCl, pH 7.2) for 48 h and then stored at 4 °C in the dark.

Solution of plasmid DNA: Plasmid DNA (pBSK+) was isolated from *Escherichia coli* DH5α bacterial cells, cultured for 16 h in LB medium (0.5% yeast extract, 1% trypton, % NaCl) under continual stirring at 150 rpm, temperature 37 °C (Heidolph Unimax 1010 shaker, Heidolph Unimax, Schwabach, Germany). Ampicilin (50 mg.cm^−3^) was used as a selection agent. Plasmid DNA was isolated by using commercial isolation kit Fast-n-Easy Plasmid Mini-Prep Kit (Jena Bioscience, Jena, Germany). The pDNA obtained in the last step of the isolation procedure was dissolved in 50 mM phosphate buffer, pH 7.2.

The concentration of DNA was determined spectrophotometrically at 260 nm on a NanoDrop spectrophotometer ND-1000 (Nanodrop Technologies, Wilmington, DE, USA). Each solution of DNA was found to be of sufficient purity as evident from the absorbance ratio values A_260_/A_230_ in the range 2.0–2.2 and A_260_/A_280_ ≥ 1.8.

### 3.3. UV-Vis Spectroscopic Studies

Complex formation between cupric ions and phenolic compounds was studied at molar ratios of 1:1 and 1:2 in DMSO-phosphate buffer (5% *v*/*v*). Phenolic compound (0.1 mM) was added to a solution containing 0.1 mM (1:1) or 0.05 mM CuCl_2_.2H_2_O (Metal-to-ligand ratio 1:2). The final total volume in the test tube was adjusted to 3 mL using DMSO and the solution was left to stand for 60 s. Subsequently, the absorption spectra of the phenolic compounds (50 μM) and the changes in their respective spectra in the presence of cupric ions were recorded in the wavelength range of 200–600 nm. The UV-Vis absorbance measurements were performed on a Cary 60 spectrophotometer (Agilent Technologies, Santa Clara, CA, USA) at room temperature (25 °C) using matched quartz cells of 1.0 cm path length.

### 3.4. EPR Spectroscopy

EPR spectra of copper-flavonoid (metal-to-ligand ratio = 1:2) DMSO solutions (1 mM) were measured using an EMX EPR spectrometer (Bruker, Karlsruhe, Germany) at 77 K at X-band (cca 9.4 GHz) using cylindrical quartz tubes [52].

### 3.5. ABTS Radical Scavenging Activity

The time dependent ABTS radical reduction activity was monitored from the decay of the absorption band of the ABTS radical cation (ABTS^•^^+^) in the sample at a fixed wavelength of 734 nm [53]. Firstly, the ABTS solution was prepared by dissolving 17.2 mg of ABTS (2,2′-azino-bis(3-ethylbenzothiazoline-6-sulphonic acid) and 3.3 mg of potassium persulfate in 5 mL of deionized water. To achieve complete oxidation of the ABTS salt to ABTS^•^^+^ the solution was left at room temperature for 24 h. Working solution of ABTS^•^^+^ were prepared by diluting 1 mL of solution containing oxidized ABTS^•^^+^ with 60 mL of deionized water. Subsequently, 2.8 mL of ABTS^•^^+^ solution was added rapidly to the test tube containing 0.2 mL of sample. The sample consisted of free phenolic compound or Cu(II)-phenolic complex in molar ratios 1:1 and 1:2 in DMSO. The kinetic of the reaction of ABTS^•^^+^ in the presence of system studied was recorded for 900 s until a steady state level was reached, which was observed as a plateau of the spectral curve. The ABTS radical cation inhibition percentage was calculated according to the reaction: Inhibition % = 100 × (A_0_ − A)/A, where A_0_ indicates the absorbance of the blank and A indicates the absorbance of the sample (free phenolic compound or phenolic compound in the presence of Cu(II) ions).

### 3.6. Electronic Absorption Titrations

CT DNA binding studies were performed by titrating a fixed concentration (0.02 mM) of compounds (free phenolic compound and Cu(II)-phenolic compound system at molar ratio of 1:2) while increasing the CT DNA concentration (10–50 μM) in TBS buffer (pH 7.2). The sample containing DNA was allowed to incubate for 10 min at room temperature before the absorption spectra were recorded. Absorption spectra for all free phenolic compounds and their mixtures with Cu(II) ions were recorded in the range 200–600 nm. A correction was made for the absorbance of the DNA itself.

The intrinsic binding constant *K_b_* (in M^−1^) of the systems studied, containing CT DNA reflects the binding strength of the mutual interaction, and was calculated from the equation [54]:

(3) $$\frac{\left[\mathrm{DNA}\right]}{\left(\varepsilon_{a}-\varepsilon_{f}\right)}=\frac{\left[\mathrm{DNA}\right]}{\left(\varepsilon_{b}-\varepsilon_{f}\right)}+\frac{1}{K_{b}\left(\varepsilon_{b}-\varepsilon_{f}\right)}$$

where [DNA] is the concentration of CT DNA in base pairs, the coefficients *ε_a_*, *ε_f_*, and *ε_b_* are the apparent absorption coefficients and correspond to A_obsd_/[studied system], the extinction coefficient of free studied system for each addition to DNA and the extinction coefficient for the bound studied system, respectively. The data obtained were plotted to give a straight line, and the *K_b_* value was calculated from the ratio of slope to the intercept.

### 3.7. Gel Electrophoresis

The reaction mixture was prepared in a total volume of 15 μL. Solutions of CuCl_2_ and of the phenolic compounds were freshly prepared and used immediately. The 5 μM CuCl_2_ and respective concentrations of phenolic compounds in DMSO (5–500 μM MYR/MOR/DHF; 5–5000 μM TAX/CUM) were mixed at room temperature. The reaction mixture was allowed to stand for 10 min prior to addition of 20 μM plasmid DNA in 50 mM sodium phosphate buffer (pH 7.2). After 5 min, 50 μM hydrogen peroxide was added and the mixture was incubated for 30 min. Along with these experiments, variants were performed under the same conditions with the selected concentrations of phenolic compounds, both alone and with cupric ions, but without the addition of hydrogen peroxide. Following the 30-min incubation, 6 μL of the quench buffer (0.25% bromphenol blue, 30% glycerol) was added and samples were immediately loaded onto 0.8% agarose gel in TBE (89 mM Tris-borate acid, 2 mM EDTA, pH = 8.0) containing 0.5 μg/mL of ethidium bromide. Electrophoresis was completed in TBE buffer for 1.5 h at 80 V. The 1 kb DNA ladder (Solis BioDyne) was used as a standard. The reference plasmid sample was linearized with EcoRI endonuclease (Thermo Fisher Scientific, Vienna, Austria) and used as a control for double strand breaks. The gel was imaged under UV light (Kodak Gel Logic 200, Eastman Kodak Company, Rochester, NY, USA). Each experiment was performed in triplicate, under the same conditions. Quantification of band intensities was performed by densitometric analysis using Kodak Gel Logic 200 software. The relative intensities of bands of the respective conformation states were normalized for each lane so that % SC + % OC + % LIN = 100% [55].

### 3.8. Identification of ROS

Radical scavenging agents, such as L-histidine (20 mM), DMSO (6 μL) and superoxide dismutase (SOD) enzyme (15U) were added to the reaction mixture prior addition of hydrogen peroxide to identify assumed formation of singlet oxygen (^1^O_2_), hydroxyl radical (^•^OH) and a superoxide radical anion (O_2_^•^^−^), respectively. Quantities of the scavengers are related to the final volume of 15 μL. Electrophoretic conditions were identical, as described above.

## 4. Conclusions

The structural characteristics of flavonoids, particularly the number and position of hydroxyl groups, planarity of the molecular skeleton, extent of delocalization and ability to chelate redox metal ions are key features affecting their antioxidant or prooxidant properties. The aim of this work was to ascertain the structural characteristics of a series of structurally different flavonoids responsible for their antioxidant and/or prooxidant properties in the presence of Cu(II) ions. The selected phenolic compounds were myricetin, morin, 3′,4′-dihydroxyflavone, taxifolin and 4-hydroxycoumarin. These substances differ in the number and positions of hydroxyl groups, the presence or absence of conjugation in the system and their ability to chelate Cu(II) ions and other structural features.

The radical scavenging activity of flavonoid molecules (ABTS assay) decreases in the order myricetin > morin > 3′,4′-dihydroxyflavone ~ coumarin > taxifolin, in agreement with the deceasing number of hydroxyl groups of flavonoids, planarity of the molecular skeleton and extent of electron delocalization. The B ring of the flavonoid skeleton is the most biologically significant molecular functionality in scavenging of ROS because it donates hydrogen and/or an electron to a variety of ROS including hydroxyl and peroxyl radicals, stabilizing them and giving rise to formation of a relatively stable flavonoid radicals. This is in agreement with the general rule that the molecules with more extended conjugated systems form resonance-stabilized free radicals with prolonged life-times and exhibit more profound radical scavenging activity.

A variety of diseases are characterized by disturbed metabolism of redox active metals such as Cu(II), thus studies of their interaction with flavonoids have pharmacological importance. Copper-chelating activity of studied flavonoids revealed that a tight interaction between phenolic compounds and Cu^2+^ ions is achieved via the 3-OH and 4-CO groups only if the unsaturated C2-C3 bond is present in the C-ring, which further permits through-conjugation with the B-ring.

The major mechanisms by which flavonoids can exert their biological effects involve the activation of apoptotic proteins, increased formation of ROS and induction of DNA damage. Several flavonoids including myricetin are known to inhibit DNA synthesis in bacteria [56]. It has been proposed that the B ring of the flavonoids may intercalate or participate in the formation of hydrogen bonds with the stacking of nucleic acid bases which in turn lead to inhibition of DNA synthesis. Interaction of phenolic compounds and their Cu(II) complexes with DNA was studied by means of absorption titrations. The results showed that myricetin can interact with Cu(II) via donor atoms in the C ring (3-OH and 4-CO groups) and the B ring (3′-OH and 4′-OH groups) and it exhibits an interaction with DNA of intermediate strength. Morin interacts with Cu(II) exclusively via donor atoms on the C ring (3-OH and 4-CO groups) and exhibits a mild interaction strength with DNA, while 3′,4′-dihydroxyflavone, which can interact exclusively via the 3′-OH and 4′-OH groups on the ring B, exhibits a strong interaction with DNA. The overall results indicate that copper ions can modulate the DNA binding affinity of flavonoids via the formation of their Cu-chelates.

From a DNA damage/protection study, by means of gel electrophoresis, it was concluded that the protective effect of the phenolic compounds studied decreases in the order: 3′,4′-dihydroxyflavone > coumarin > morin > taxifolin ~ myricetin. The results clearly show that more pronounced DNA damage occurs in the presence of those flavonoids containing higher number of hydroxyl groups including 3-OH group and involve myricetin (six hydroxyl groups), morin and taxifolin (five hydroxyl groups). All these flavonoids form stable Cu(II) chelates capable of causing DNA damage via their interaction with DNA and the formation of ROS via the Fenton reaction. An application of ROS scavengers documented formation of singlet oxygen, superoxide radical anion and hydroxyl radicals. An analysis of electrophoretic profiles revealed that the concerted action of a variety of ROS synergistically enhances DNA damage.

Based on the obtained results we may conclude that the flavonoids may behave as both antioxidants or prooxidants, depending on the conditions such as presence of redox active Cu(II) ions. Recently reported cytotoxicity studies have revealed that anticancer activity of flavonoids may be related to their prooxidant mechanism of action [57]. Cancer cells are well adapted to higher level of oxidative stress compared to normal cells, rendering malignant cells more vulnerable to being killed by drugs such as flavonoids alone or their metal- (Cu-) flavonoid complexes that boost ROS levels in cancer cells [51]. Whether a flavonoid will act as an antioxidant or a prooxidant depends on its dose, stability of metal-flavonoid complex and number of hydroxyl groups. The sensitivities of cancer cells against flavonoids or their metal-complexes may differ depending on the type of tissue, indicating that the flavonoid-induced cytotoxicity might be related to certain types of cancers [57].

Overall, these results suggest that the higher number of hydroxyl groups including 3-OH group of the C ring of phenolic compounds is critical for the induction of DNA damage in the presence of cupric ions. There is the further indication that the mechanism by which myricetin, morin and taxifolin and their Cu(II) complexes can interact with DNA, predisposes these substances to act as potential anticancer agents [48]. The potential anticancer properties of phenolic compounds are related to their moderate prooxidant activity, which can boost formation of ROS and kill cancer cells. Alternatively, as a prevention strategy, slight prooxidant activity of flavonoids may induce cellular antioxidant systems, including antioxidant enzymes and synthesis of low molecular antioxidants such as glutathione and thus act as preventive anticancer therapeutic agents [58,59,60,61,62,63,64].

## Figures and Tables

**Table 1:** Inhibition [%] of radical cation of ABTS (ABTS^•+^) by phenolic compounds (PhC).

% Inhibition					
	MYR	MOR	DHF	TAX	CUM
PhC	98.10 ± 1.42	74.05 ± 0.41	36.56 ± 0.77	21.79 ± 0.71	35.67 ± 0.77
Cu:PhC (1:1)	81.50 ± 1.48	69.22 ± 1.36	38.02 ± 1.12	23.71 ± 0.34	36.62 ± 1.20
Cu:PhC (1:2)	79.66 ± 1.76	60.59 ± 1.66	38.82 ± 1.32	29.19 ± 0.45	36.28 ± 1.13

PhC = Phenolic compound; MYR = Myricetin; MOR = Morin; DHF = 3′,4′-dihydroxyflavone; TAX = Taxifolin; CUM = 4-hydroxycoumarin. Concentration of phenolic compounds = 8.33 µmol.

**Table 2:** Overview of wavelength values of absorption peaks for free phenolic compounds and their chelates with copper ions, percentages of hypochromic (↓) and hyperchromic (↑) shifts. Bathochromic and hypsochromic shifts are denoted (**→**) and (**←**), respectively and are expressed in nm.

Phenolic Compound	Band 1	↓↑ % →/← nm	Band 2	↓↑ % →/← nm	Band 3	↓↑ % →/← nm
MYR	267 nm	↓ 45.95 → 8 nm	322 nm	↑ 23.33 → 10 nm	378 nm	↓ 72.97 ← 5 nm
Cu-MYR	267 nm	↓ 36.51 ← 5 nm	346 nm	↑ 23.33 ← 21 nm	441 nm	↓ 66.67 → 2 nm
MOR	269 nm	↓ 22.86 -	393 nm	↓ 21.64 -		
Cu-MOR	269 nm	↓ 42.25 → 7 nm	330 nm	↑ 21.62 -	398 nm	↓ 55.17 ← 2 nm
DHF	244 nm	↓ 17.86 ← 2 nm	342 nm	↓ 22.83 -		
Cu-DHF	281 nm	↓ 26.83 -	308 nm	↓ 28.00 -	397 nm	↓ 31.75 -
TAX	326 nm	↓ 25.37 -				
Cu-TAX	326 nm	↓ 27.59 -				
CUM	287 nm	↓ 20.59 -				

**Table 3:** Binding constants (K_b_) of free phenolic compounds (FL) with DNA, and in the presence of cupric ions (Cu:FL = 1:2).

Flavonoid	MYR	MOR	DHF	TAX	CUM
FL	2.07 × 10^4^	3.23 × 10^4^	2.43 × 10^4^	1.44 × 10^4^	6.61 × 10^4^
Cu:FL (1:2)	4.02 × 10^4^	8.08 × 10^3^	1.23 × 10^5^	-	-
